# Predicting Classifier Performance with Limited Training Data: Applications to Computer-Aided Diagnosis in Breast and Prostate Cancer

**DOI:** 10.1371/journal.pone.0117900

**Published:** 2015-05-18

**Authors:** Ajay Basavanhally, Satish Viswanath, Anant Madabhushi

**Affiliations:** Department of Biomedical Engineering, Case Western Reserve University, Cleveland, OH, USA; Princess Margaret Cancer Centre, CANADA

## Abstract

Clinical trials increasingly employ medical imaging data in conjunction with supervised classifiers, where the latter require large amounts of training data to accurately model the system. Yet, a classifier selected at the start of the trial based on smaller and more accessible datasets may yield inaccurate and unstable classification performance. In this paper, we aim to address two common concerns in classifier selection for clinical trials: (1) predicting expected classifier performance for large datasets based on error rates calculated from smaller datasets and (2) the selection of appropriate classifiers based on expected performance for larger datasets. We present a framework for comparative evaluation of classifiers using only limited amounts of training data by using random repeated sampling (RRS) in conjunction with a cross-validation sampling strategy. Extrapolated error rates are subsequently validated via comparison with leave-one-out cross-validation performed on a larger dataset. The ability to predict error rates as dataset size increases is demonstrated on both synthetic data as well as three different computational imaging tasks: detecting cancerous image regions in prostate histopathology, differentiating high and low grade cancer in breast histopathology, and detecting cancerous metavoxels in prostate magnetic resonance spectroscopy. For each task, the relationships between 3 distinct classifiers (k-nearest neighbor, naive Bayes, Support Vector Machine) are explored. Further quantitative evaluation in terms of interquartile range (IQR) suggests that our approach consistently yields error rates with lower variability (mean IQRs of 0.0070, 0.0127, and 0.0140) than a traditional RRS approach (mean IQRs of 0.0297, 0.0779, and 0.305) that does not employ cross-validation sampling for all three datasets.

## Introduction

A growing amount of clinical research employs computerized classification of medical imaging data to develop quantitative and reproducible decision support tools [1–3]. A key issue during the development of image-based classifiers is the accrual of sufficient data to achieve a desired level of statistical power and, hence, confidence in the generalizability of the results. Computerized image analysis systems typically involve a supervised classifier that needs to be trained on a set of annotated examples, which are often provided by a medical expert who manually labels the samples according to their disease class (e.g. high or low grade cancer) [4]. Unfortunately, in many medical imaging applications, accumulating large cohorts is very difficult due to (1) the high cost of expert analysis and annotations and (2) because of overall data scarcity [3, 5]. Hence, the ability to predict the amount of data required to achieve a desired classification accuracy for large-scale trials, based on experiments performed on smaller pilot studies is vital to the successful planning of clinical research.

Another issue in utilizing computerized image analysis for clinical research is the need to select the best classifier at the onset of a large-scale clinical trial [6]. The selection of an optimal classifier for a specific dataset usually requires large amounts of annotated training data [7] since the error rate of a supervised classifier tends to decrease as training set size increases [8]. Yet, in clinical trials, this decision is often based on the assumption (which may not necessarily hold true [9]) that the relative performance of classifiers on a smaller dataset will remain the same as more data becomes available.

In this paper, we aim to overcome the major constraints on classifier selection in clinical trials that employ medical imaging data, namely (1) the selection of an optimal classifier using only a small subset of the full cohort and (2) the prediction of long-term performance in a clinical trial as data becomes available sequentially over time.

To this end, we aim to address crucial questions that arise early in the development in a classification system, namely:

- Given a small pilot dataset, can we predict the error rates associated with a classifier assuming that a larger data cohort will become available in the future?
- Will the relative performance between multiple classifiers hold true as data cohorts grow larger?

**Fig 1:**
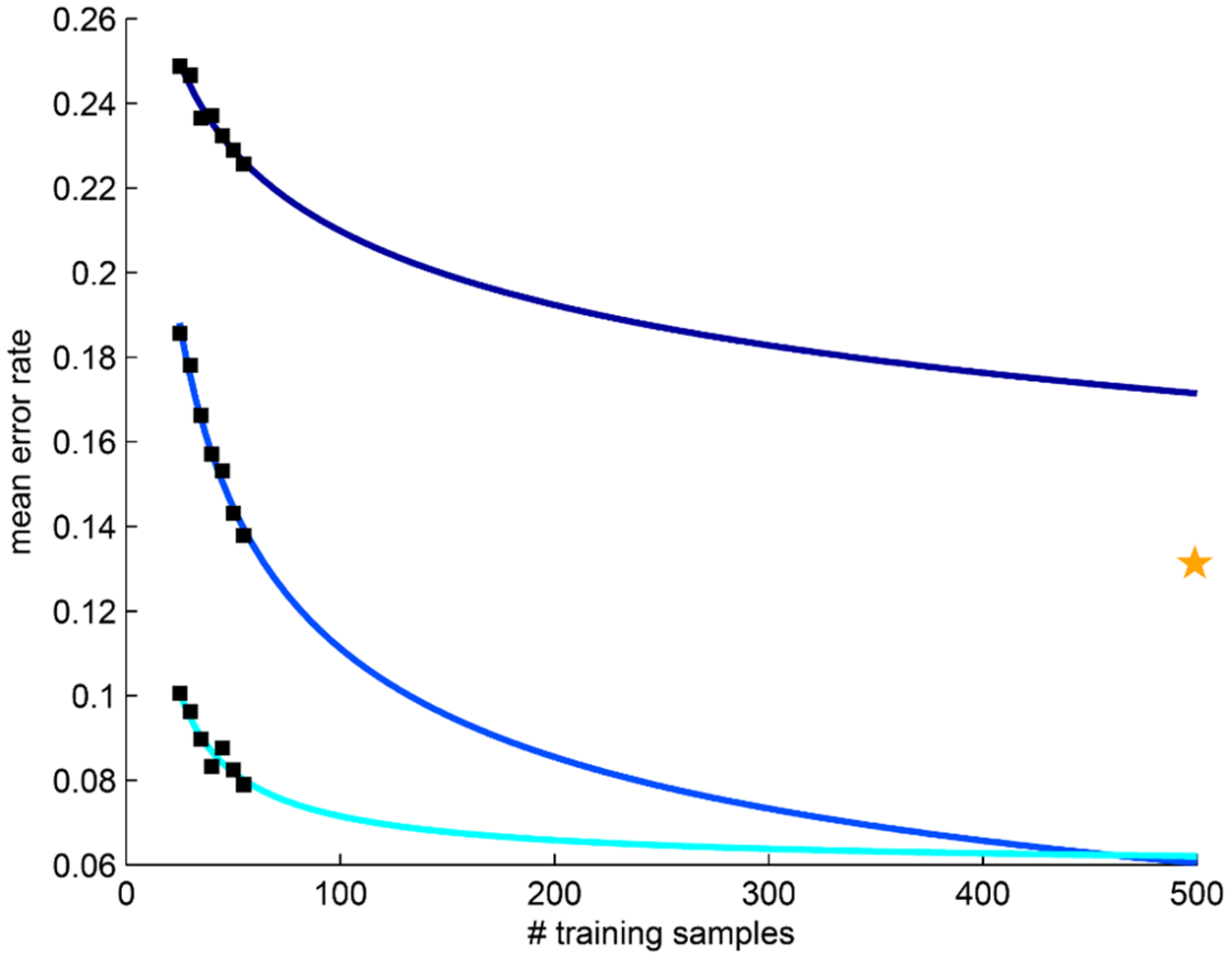
Traditional repeated random sampling (RRS) of prostate cancer histopathology leads to unstable estimation of error rates. Application of RRS to the classification of cancerous and non-cancerous prostate cancer histopathology (dataset 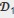) suggests that heterogeneous medical imaging data can produce highly variable calculated (black boxes) and extrapolated (blue curves) mean error rates. Each set of error rates is derived from and independent RRS trial that employs different training and testing pools for classification. The yellow star represents the leave-one-out cross-validation error (i.e. the expected lower bound on error) produced by a larger validation cohort.

Traditional power calculations aim to determine confidence in an error estimate using repeated classification experiments [10], but do not address the question of how error rate changes as more data becomes available. Also, they may not be ideal for analyzing biomedical data because they assume an underlying Gaussian distribution and independence between variables [11]. Repeated random sampling (RRS) approaches, which characterize trends in classification performance via repeated classification using training sets of varying sizes, have thus become increasingly popular, especially for extrapolating error rates in genomic datasets [6, 11, 12]. Drawbacks of RRS include (1) no guarantee that all samples will be selected at least once for testing and (2) a large number of repetitions required to account for the variability associated random sampling. In particular, traditional RRS may suffer in the presence of highly heterogeneous datasets (e.g. biomedical imaging data [13]) due to the use of fixed training and testing pools. This is exemplified in Fig 1 by the variability in calculated (black boxes) and extrapolated (blue curves) error rates resulting from the use of different training and testing pools from the same dataset. More recently, methods such as repeated independent design and test (RIDT) [14] have aimed to improve RRS by simultaneously modeling the effects of different testing set sizes in addition to different training set sizes. This approach, however, requires allocation of larger testing sets than RRS, thereby reducing the number of samples available in the training set for extrapolation. It is important to note that the concept of predicting error rates for large datasets should not confused with semi-supervised learning techniques, e.g. active learning (AL) [15], that aim to maximize classifier performance while mitigating the costs of compiling large annotated training datasets [5]. Since AL methods are designed to optimize classification accuracy during the acquisition of new data, they are not appropriate for *a priori* prediction of classifier performance using only a small dataset.

**Fig 2:**
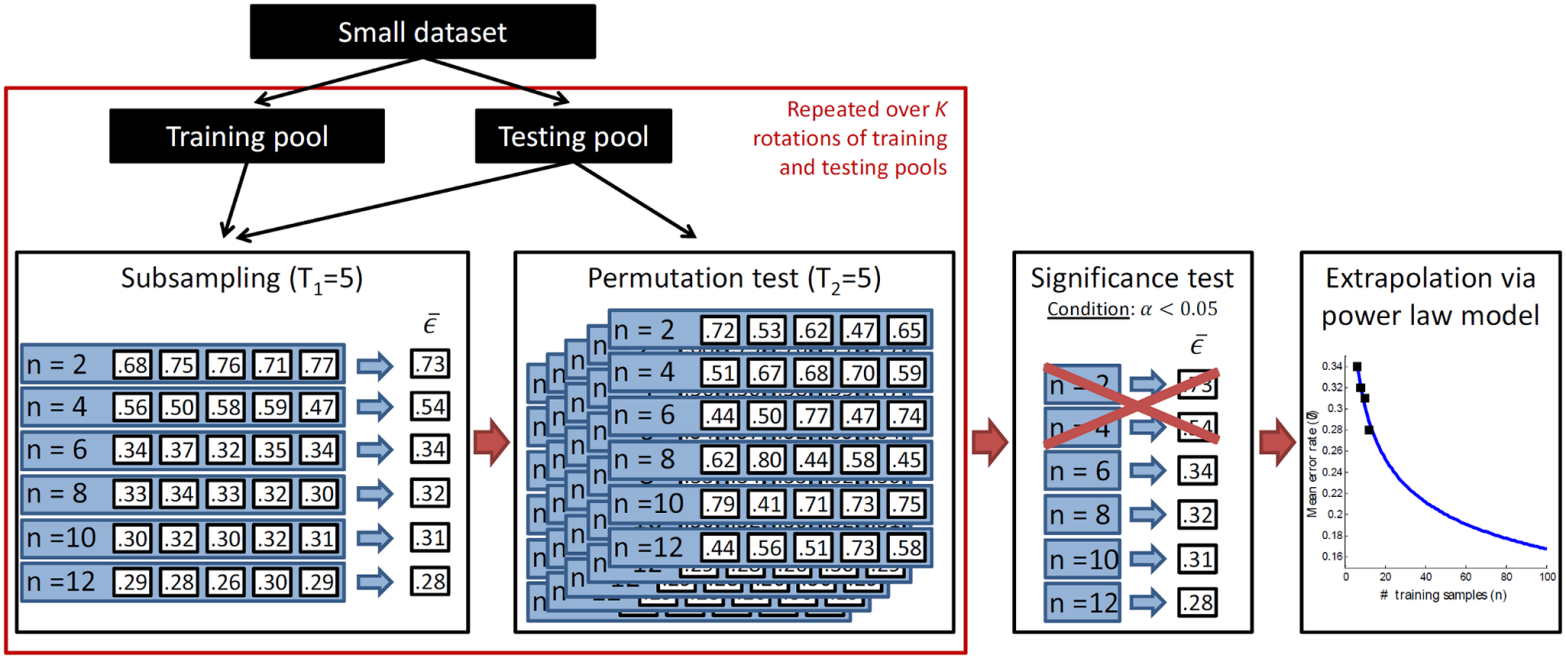
A flowchart describing the methodology used in this paper. First, a dataset is partitioned into training and testing pools using a *K*-fold sampling strategy (red box). Each of the *K* training pools undergoes traditional repeated random sampling (RRS), in which error rates are calculated at different training set sizes *n* via a subsampling procedure. A permutation test is used to identify statistically significant error rates, which are then used to extrapolate learning curves and predict error rates for larger datasets. The extension to pixel-level data employs the same sampling and error rate estimation strategies shown in this flowchart; however, the classifiers used for calculating the relevant error rates are trained and evaluated on pixel-level features from the training sets and testing pool, respectively.

Due to the heterogeneity present in biomedical imaging data, we extend the RRS-based approach originally used to model gene microarray data [11] by incorporating a *K*-fold cross-validation framework to ensure that all samples are used for both classifier training and testing (Fig 2). First, the dataset is split into *K* distinct, stratified pools where one pool is used for testing while the remaining *K* − 1 are used for training. A bootstrap subsampling procedure is used to create multiple subsets of various sizes from the training pool. Each subset is used to train a classifier, which is then evaluated against the testing pool. The pools are rotated *K* times to ensure that all samples are evaluated once and error rates are averaged for each training set size. The resulting mean error rates are used to determine the three parameters of the power-law model (rate of learning, decay rate, and Bayes error) that characterize the behavior of error rate as a function of training set size.

Application of the RRS model to patient-level medical imaging data, where each patient or image is described by a single set of features, is relatively well-understood. Yet disease classification in radiological data (e.g. MRI) occurs at the pixel-level, in which each patient has pixels from both classes (e.g. diseased and non-diseased states) and each pixel is characterized by a set of features [16]. In this work, we present an extension to RRS that employs two-stage sampling in order to mitigate the sampling bias occurring from high intra-patient correlation between pixels. The first stage requires all partitioning of the dataset to be performed at the patient-level, ensuring that pixels from a single patient will not be included in both the training and testing sets. In the second stage, pixel-level classification is performed by training the classifier using pixels from all images (and both classes) in the training set and evaluating against pixels from all images in the testing set. The resulting error rates are used to extrapolate classifier performance as previously described for the traditional patient-level RRS.

This paper focuses on comparing the performance of three exemplar classifiers: (1) the non-parametric *k*-nearest neighbor (kNN) classifier [8], (2) the probabilistic naive Bayes (NB) classifier that assumes an underlying Gaussian distribution [8], and (3) a non-probabilistic Support Vector Machine (SVM) classifier that aims to maximize class separation using a radial basis function (RBF) kernel. Each of these classifiers has previously been used for a variety of computerized image analysis tasks in the context of medical imaging [17, 18]. All classifiers are evaluated on three distinct classification problems: (1) detection of cancerous image regions in prostate cancer (PCa) histopathology [5], (2) grading of cancerous nuclei in breast cancer (BCa) histopathology [19], and (3) detection of cancerous metavoxels on PCa magnetic resonance spectroscopy (MRS) [16].

The novel contributions of this work include (1) more stable learning curves due to the incorporation of cross-validation into the RRS scheme, (2) a comparison of performance across multiple classifiers as dataset size increases, and (3) enabling a power analysis of classifiers operating on the pixel/voxel level (as opposed to patient/sample level), which cannot be currently done via standard sample power calculators.

The remainder of the paper is organized as follows. First, the **Methods** section presents a description of the methodology used in this work. **Experimental Design** includes a description of the datasets and experimental parameters used for evaluation. **Results and Discussion** are subsequently presented for all experiments, followed by **Concluding Remarks**.

## Methods

### Notation

For all experiments, a dataset 𝓓 is divided into independent training 𝓝 ⊂ 𝓓 and a testing 𝓣 ⊂ 𝓓 pools, where 𝓝∩𝓣 = ∅. The class label of a sample *x* ∈ 𝓓 is denoted by *y*_*t*_ ∈ {*ω*_1_, *ω*_2_}. A set of training set sizes **N** = {*n*_1_, *n*_2_, …, *n*_*N*_}, where 1 ≤ *n* ≤ |𝓝| and |·| denotes set cardinality.

### Subsampling test to calculate error rates for multiple training set sizes

The estimation of classifier performance first requires the construction of multiple classifiers trained on repeated subsampling of the limited dataset. For each training set size *n* ∈ **N**, a total of *T*_1_ subsets 𝓢(*n*) ⊂ 𝓝, each containing *n* samples, are created by randomly sampling the training pool 𝓝. For each *n* ∈ **N** and *i* ∈ {1, 2, …, *T*_1_}, the subset *S*_*i*_(*n*) ∈ 𝓢 is used to train a corresponding classifier *H*_*i*_(*n*). Each *H*_*i*_(*n*) is evaluated on the entire testing set 𝓣 to produce an error rate *e*_*i*_(*n*). The mean error rate for each *n* ∈ **N** is calculated as

$$\begin{array}{c} \bar{e}\left(n\right)=\frac{1}{T_{1}}\sum_{i}^{T_{1}}e_{i}\left(n\right). \end{array}$$ (1)

### Permutation test to evaluate statistical significance of error rates

Permutation tests are a well-established, non-parametric approach for implicitly determining the null distribution of a test statistic and are primarily employed in situations involving small training sets that contain insufficient data to make assumptions about the underlying data distribution [11, 20]. In this work, the null hypothesis states that the performance of the actual classifier is similar to “intrinsic noise” of a randomly trained classifier. Here, a randomly trained classifier is modeled through repeated calculation of error rates from classifiers trained on data with randomly selected class labels.

To ensure the statistical significance of the mean error rates $\bar{e}(n)$ calculated in Eq 1, the performance of training set *S*_*i*_(*n*) is compared against the performance of randomly labeled training data. For each *S*_*i*_(*n*) ∈ 𝓢(*n*), a total of *T*_2_ subsets $\hat{𝓢}(n)\subset 𝓝$, each containing *n* samples, are created. For each *n* ∈ **N**, *i* ∈ {1, 2, …, *T*_1_}, and *j* ∈ {1, 2, …, *T*_2_}, the subset ${\hat{S}}_{i,j}(n)\in \hat{𝓢}$ is assigned a randomized class label *y*_*r*_ ∈ {*ω*_1_, *ω*_2_} and used to train a corresponding classifier ${\hat{H}}_{i,j}(n)$. Each ${\hat{H}}_{i,j}(n)$ is evaluated on the entire testing set 𝓣 to produce an error rate ${\hat{e}}_{i,j}(n)$. For each *n*, a p-value

$$\begin{array}{c} P_{n}=\frac{1}{T_{1}}\frac{1}{T_{2}}\sum_{i=1}^{T_{1}}\sum_{j=1}^{T_{2}}\theta \left(\bar{e}\left(n\right)-{\hat{e}}_{i,j}\left(n\right)\right), \end{array}$$ (2)

where *θ*(*z*) = 1 if *z* ≥ 0 and 0 otherwise. *P*_*n*_ is calculated as the fraction of randomly-labeled classifiers ${\hat{H}}_{i,j}(n)$ with error rates ${\bar{e}}_{i,j}(n)$ exceeding the mean error rate $\bar{e}(n),\forall n\in \text{N}$. The mean error rate $\bar{e}(n)$ is deemed to be valid for model-fitting only if *P*_*n*_ < 0.05, i.e. there is a statistically significant difference between $\bar{e}(n)$ and $\left\{{\hat{e}}_{i,j}(n),\forall i\in \{1,2,\ldots ,T_{1}\},\forall j\in \{1,2,\ldots ,T_{2}\}\right\}$. Hence, the set of valid training set sizes **M** = {*n*:*n* ∈ **N**, *P*_*n*_ < 0.05} includes only those *n* ∈ **N** that have passed the significance test.

### Cross-validation strategy for selection of training and testing pools

The selection of training 𝓝 and testing 𝓣 pools from the limited dataset 𝓓 is governed by a *K*-fold cross-validation strategy. In this paper, the dataset 𝓓 is partitioned into *K* = 4 pools in which one pool is used for evaluation while the remaining *K* − 1 pools are used for training to produce mean error rates ${\bar{e}}_{k}(n)$, where *k* ∈ {1, 2, …, *K*}. The pools are then rotated and the subsampling and permutation tests are repeated until all pools have been evaluated exactly once. This process is repeated over *R* cross-validation trials, yielding mean error rates ${\bar{e}}_{k,r}(n)$ where *r* ∈ {1, 2, …, *R*}. For all training set sizes that have passed the significance test, i.e. ∀*n* ∈ **M**, learning curves are generated from a comprehensive mean error rate

$$\begin{array}{c} \bar{e}\left(n\right)=\frac{1}{K}\frac{1}{R}\sum_{k=1}^{K}\sum_{r=1}^{R}{\bar{e}}_{k,r}\left(n\right), \end{array}$$ (3)

calculated over all cross-validation folds *k* ∈ {1, 2, …, *K*} and iterations *r* ∈ {1, 2, …, *R*}.

### Estimation of power law model parameters

The power-law model [11] describes the relationship between error rate and training set size

$$\begin{array}{c} \bar{e}\left(n\right)=an^{-\alpha }+b, \end{array}$$ (4)

where $\bar{\text{e}}(n)$ is the comprehensive mean error rate (Eq 3) for training set size *n*, *a* is the learning rate, and *α* is the decay rate. The Bayes error rate *b* is defined as the lowest possible error given an infinite amount of training data [8]. The model parameters *a*, *α*, and *b* are calculated by solving the constrained non-linear minimization problem

$$\begin{array}{c} \min_{a,\alpha ,b}\sum_{m=1}^{\left|M\right|}{\left(an_{m}S^{-\alpha }+b-\bar{e}\left(n\right)\right)}^{2}, \end{array}$$ (5)

where *a*, *α*, *b* ≥ 0.

### Extension of error rate prediction to pixel- and voxel-level data

The methodology presented in this work can be extended to such pixel- or voxel-level data by first selecting training set sizes **N** at the patient-level. Definition of the *K* training and testing pools as well as creation of each subsampled training set *S*_*i*_(*n*) ∈ 𝓢 are also performed at the patient-level. Training of the corresponding classifier *H*_*i*_(*n*), however, is performed at the pixel-level by aggregating pixels for all patients in *S*_*i*_(*n*). A similar aggregation is done for all patients in the testing pool 𝓣. By ensuring that all pixels from a given patient remain together, we are able to perform pixel-level calculations while avoiding the sampling bias that occurs when pixels from a single patient span both training and testing sets.

## Experimental Design

Our methodology is evaluated on a synthetic dataset and 3 actual classification tasks traditionally affected by limitations in the availability of imaging data (Table 1). All experiments have a number of parameters in common, including *T*_1_ = 50 subsampling trials, *T*_2_ = 50 permutation trials, and *R* = 10 independent trials of *K* = 4 fold cross-validation. In addition, all experiments employ the *k*-nearest neighbor (kNN), naive Bayes (NB), and Support Vector Machine (SVM) classifiers. A more detailed description of each classifier is presented in S1 Appendix. In each experiment, validation is performed via leave-one-out (LOO) classification on a larger dataset, which allows us to maximize the number of training samples used for classification while yielding the expected lower bound of the error rate.

**Table 1:** List of the breast cancer and prostate cancer datasets used in this study.

Notation	Description	# train. samples	# valid. samples
𝓓_1_	Prostate: Cancer detection on histopathology	100	500
𝓓_2_	Breast: Cancer grading on histopathology	46	116
𝓓_3_	Prostate: Cancer detection on MRS	16	34

For 𝓓_1_ and 𝓓_2_, each sample is treated independently during the selection of training and testing sets. For 𝓓_3_, training and testing sets are selected at the patient-level, while classification is performed at the metavoxel-level by using all metavoxels from both classes for a specified patient.

### Ethics Statement

The three different datasets used in this study were retrospectively acquired from independent patient cohorts, where the data was initially acquired under written informed consent at each collecting institution. All 3 datasets comprised de-identified medical image data and provided to the authors through the IRB protocol # E09-481 titled “Computer-Aided Diagnosis of Cancer” and approved by the Rutgers University Office of Research and Sponsored Programs. Further written informed consent was waived by the IRB board, as all data was being analyzed retrospectively, after de-identification.

### Experiment 1: Identifying cancerous tissue in prostate cancer histopathology

Automated systems for detecting PCa on biopsy specimens have the potential to act as (1) a triage mechanism to help pathologists spend less time analyzing samples without cancer and (2) an initial step for decision support systems that aim to quantify disease aggressiveness via automated Gleason grading [5]. Dataset 𝓓_1_ comprises anonymized hematoxylin and eosin (H & E) stained needle-core biopsies of prostate tissue digitized at 20x optical magnification on a whole-slide digital scanner. Regions corresponding to PCa were manually delineated by a pathologist and used as ground truth. Slides were divided into non-overlapping 30 × 30-pixel tissue regions and converted to a grayscale representation. A total of 927 features including first-order statistical, Haralick co-occurrence [21], and steerable Gabor filter features were extracted from each image [22] (Table 2). Due to the small number of training samples used in this study, the feature set was first reduced to two descriptors via the minimum redundancy maximum relevance (mRMR) feature selection scheme [23], primarily to avoid the curse of dimensionality [8]. A relatively small dataset of 100 image regions, with training set sizes **N** = {25, 30, 35, 40, 45, 50, 55}, was used to extrapolate error rates (Table 1). LOO cross-validation was subsequently performed on a larger dataset comprising 500 image regions.

**Table 2:** A summary of all features extracted from prostate cancer histopathology images in dataset 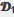. All textural features were extracted separately for red, green, and blue color channels.

Features	Parameters
Texture: Gray-level (Average, Median, Standard Deviation, Range, Sobel, Kirsch, Gradient, Derivative)	window sizes: {3, 5, 7}
Texture: Haralick co-occurrence (Joint Entropy, Energy, Inertia, Inverse Difference Moment, Correlation, Measurements of Correlation, Sum Average, Sum Variance, Sum Entropy, Difference Average, Difference Variance, Difference Entropy, Shade, Prominence, Variance)	window sizes: {3, 5, 7}
Texture: steerable Gabor filter responses (cosine and sine components combined)	window sizes: {3, 5, 7} frequency shift: {0, 1, …, 7} orientations: $\left\{0,\frac{\pi }{8},\frac{2\pi }{8},\ldots ,\frac{7\pi }{8}\right\}$

### Experiment 2: Distinguishing high and low tumor grade in breast cancer histopathology

**Fig 3:**
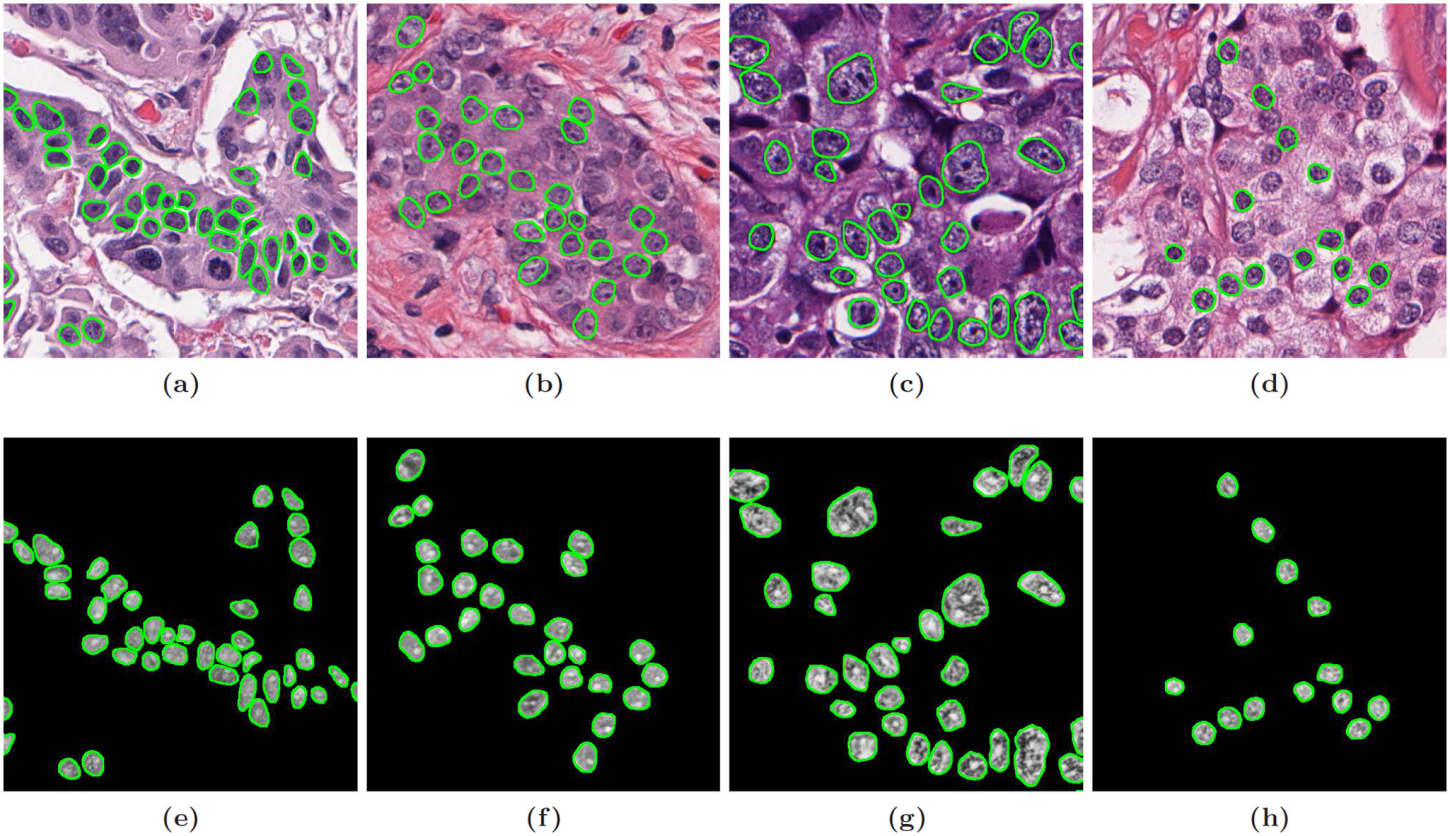
Breast cancer (BCa) histopathology images from dataset 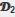. Examples of (a), (b) low modified-Bloom-Richardson (mBR) grade and (c), (d) high mBR grade images are shown with boundary annotations (green outline) for exemplar nuclei. A variety of morphological and textural features are extracted from the nuclear regions, including (e)-(h) the Sum Variance Haralick textural response.

Nottingham, or modified Bloom-Richardson (mBR), grade is routinely used to characterize tumor differentiation in breast cancer (BCa) histopathology [24]; yet, it is known to suffer from high inter- and intra-pathologist variability [25]. Hence, researchers have aimed to develop quantitative and reproducible classification systems for differentiating mBR grade in BCa histopathology [19]. Dataset 𝓓_2_ comprises 2000 × 2000 image regions taken from anonymized H & E stained histopathology specimens of breast tissue digitized at 20x optical magnification on a whole-slide digital scanner. Ground truth for each image was determined by an expert pathologist to be either low (mBR < 6) or high (mBR > 7) grade. First, boundaries of 30–40 representative epithelial nuclei were manually segmented in each image region (Fig 3). Using the segmented boundaries, a total of 2343 features were extracted from each nucleus to quantify both nuclear morphology and nuclear texture (Table 3). A single feature vector was subsequently defined for each image region by calculating the median feature values of all constituent nuclei. Similar to Experiment 1, mRMR feature selection was used to isolate the two most important descriptors. Error rates were extrapolated from a small dataset comprising 45 images with training set sizes **N** = {20, 22, 24, 26, 28, 30, 32}, while LOO cross-validation was subsequently performed on a larger dataset comprising 116 image regions (Table 1).

**Table 3:** A summary of all features extracted from breast cancer histopathology images in dataset 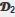. All textural features were extracted separately for red, green, and blue color channels from the RGB color space and the hue, saturation, and intensity color channels from the HSV color space.

Features	Parameters
Morphological: Basic (Area, Major Axis Length, Minor Axis Length, Eccentricity, Convex Area, Filled Area, Equivalent Diameter, Solidity, Extent, Perimeter, Area Overlap, Average Radial Ratio, Compactness, Convexity, Smoothness, Std. Dev. of Distance Ratio)	–
Morphological: Fourier Descriptors	orientations: $\left\{0,\frac{\pi }{6},\frac{2\pi }{6},\ldots ,\frac{5\pi }{6}\right\}$
Texture: Gray-level (Average, Median, Standard Deviation, Range, Sobel, Kirsch, Gradient, Derivative)	window sizes: {3, 5, 7}
Texture: Local binary patterns	window size: 3 offsets: {0, 1, …, 7} directions: clockwise, counter-clockwise
Texture: Laws (pairwise convolution of Level, Edge, Spot, Wave, Ripple filters)	–
Texture: steerable Gabor filter responses (cosine and sine components are separate features)	window sizes: {3, 5, 9} orientations: $\left\{0,\frac{\pi }{12},\frac{2\pi }{12},\ldots ,\frac{6\pi }{12}\right\}$

### Experiment 3: Identifying cancerous metavoxels in prostate cancer magnetic resonance spectroscopy

**Fig 4:**
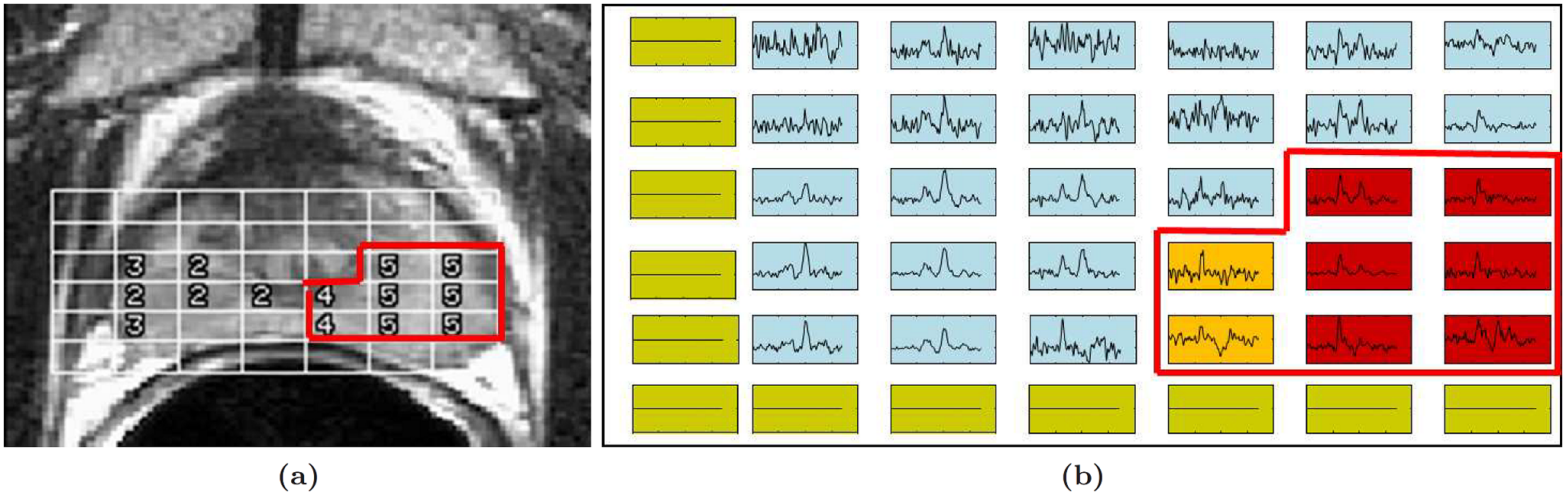
Magnetic resonance spectroscopy (MRS) data from dataset 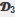. (a) A study from dataset 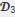 showing an MR image of the prostate with MRS metavoxel locations overlaid. (b) For ground truth, each MRS spectrum is labeled as either cancerous (red and orange boxes) or benign (blue boxes). Green boxes correspond to metavoxels outside the prostate for which MRS spectra were suppressed during acquisition.

Magnetic resonance spectroscopy (MRS), a metabolic non-imaging modality that obtains the metabolic concentrations of specific molecular markers and biochemicals in the prostate, has previously been shown to supplement magnetic resonance imaging (MRI) in the detection of PCa [16, 26]. These include choline, creatine, and citrate, and changes in their relative concentrations (choline/citrate or [choline+creatine)/citrate], which have been shown to be linked to presence of PCa [27]. Radiologists typically assess presence of PCa on MRS by comparing ratios between choline, creatine, and citrate peaks to predefined normal ranges. Dataset 𝓓_3_ comprises 34 anonymized 1.5 Tesla T2-weighted MRI and MRS studies obtained prior to radical prostatectomy, where the ground truth was defined (as cancer and benign metavoxels) via visual inspection of MRI and MRS by an expert radiologist [16] (Fig 4). Six MRS features were defined for each metavoxel by calculating expression levels for each metabolite as well as ratios between each pair of metabolites. Similar to Experiment 1, mRMR feature selection was used to identify the two most important features in the dataset. Error rates were extrapolated from a dataset of 16 patients using training set sizes **N** = {2, 4, 6, 8, 10, 12}, followed by LOO cross-validation on a larger dataset of 34 patients (Table 1).

### Comparison with traditional RRS via interquartile range (IQR)

This experiment compares the results of Experiment 1 with the traditional RRS approach, using both dataset 𝓓_1_ and corresponding experimental parameters from Experiment 1. However, since traditional RRS does not use cross-validation, a total of ${\hat{T}}_{1}=T_{1}\cdot K\cdot R$ subsampling procedures are used to ensure that same number of classification tasks are performed for both approaches. Evaluation is performed via (1) comparison of the learning curves between the two methods and (2) the interquartile range (IQR), a measure of statistical variability defined as the difference between the 25th and 75th percentile error rates from the subsampling procedure.

### Synthetic experiment using pre-defined data distributions

**Fig 5:**
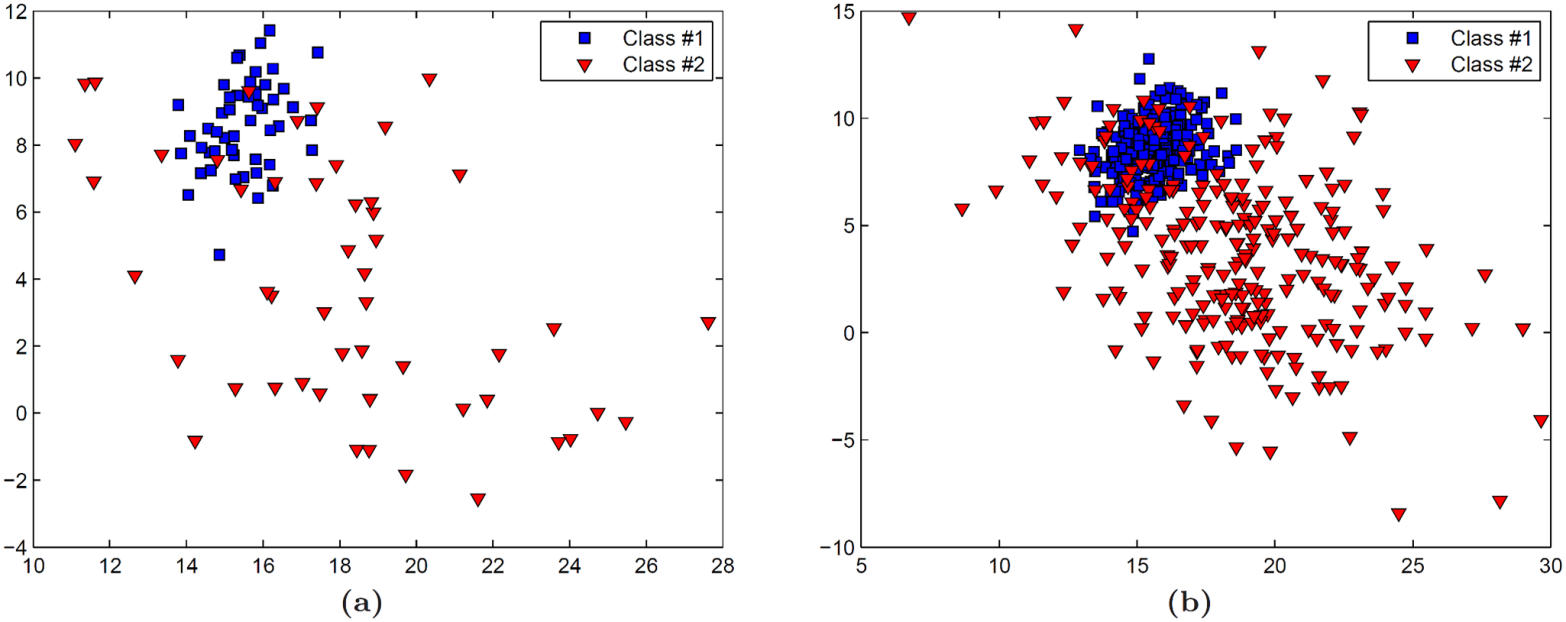
A synthetic dataset is used to validate our cross-validated repeated random sampling (RRS) method. In this dataset, each class is defined by samples drawn randomly from an independent two-dimensional Gaussian distribution. (a) A small set comprising 100 samples is used for creation of the learning curves and (b) a larger set comprising 500 samples is used for validation.

The ability of our approach to produce accurate learning curves with low variance was evaluated using a 2-class synthetic dataset, in which each class is defined by randomly selected samples from a two-dimensional Gaussian distribution (Fig 5). Learning curves are created from a small dataset comprising 100 samples (Fig 5(a)) using training set sizes **N** = {25, 30, 35, 40, 45, 50, 55} in conjunction with a kNN classifier. Validation is subsequently performed on a larger dataset containing 500 samples (Fig 5(b)).

## Results and Discussion

### Experiment 1: Distinguishing cancerous and non-cancerous regions in prostate histopathology

**Fig 6:**
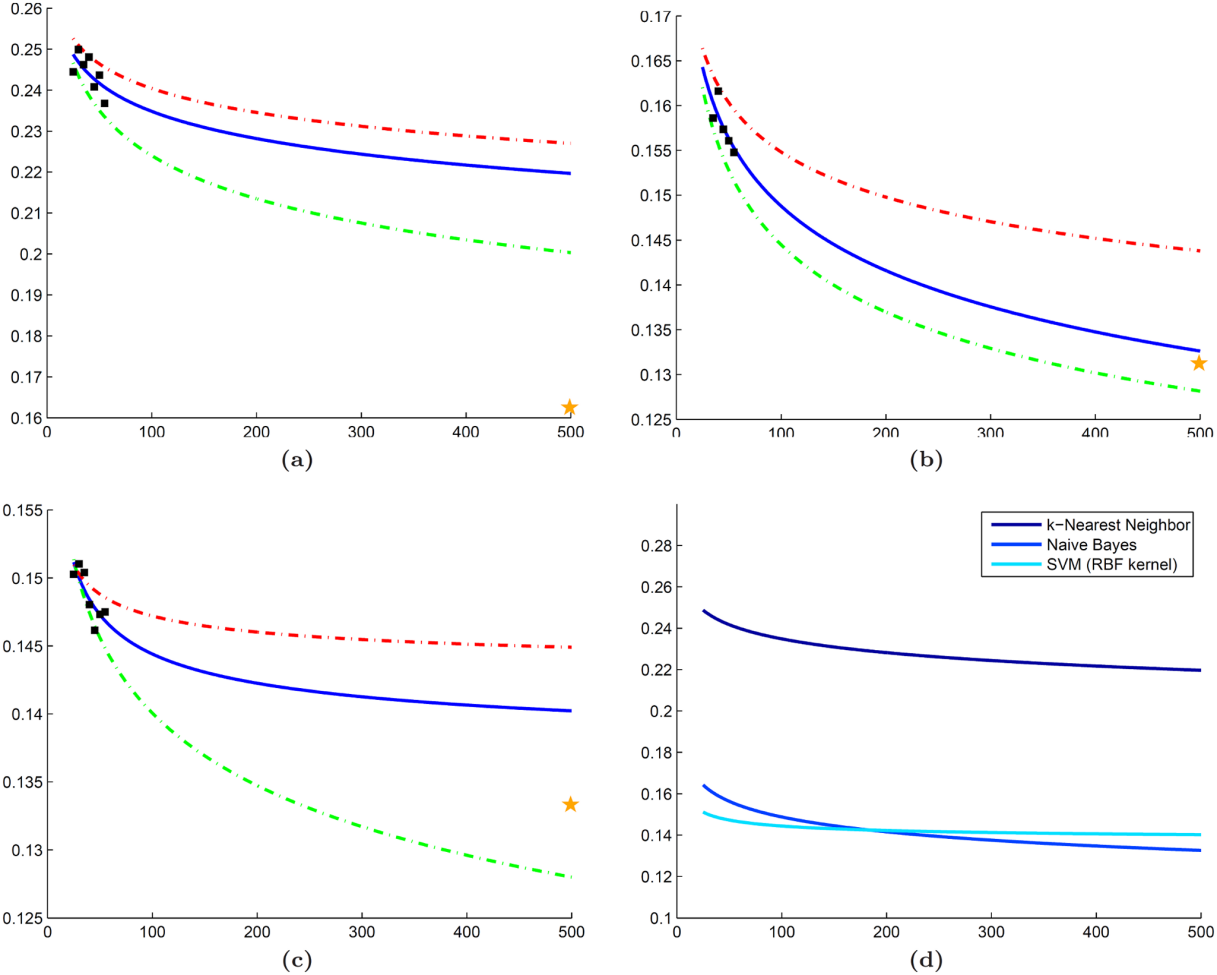
Experimental results for dataset 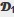. Learning curves (blue line) generated for dataset 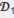 using mean error rates (black squares) calculated from (a) kNN, (b) NB, and (c) SVM classifiers. Each classifier is accompanied by curves for the 25th (green dashed line) and 75th (red dashed line) percentile of the error as well as LOO error on the validation cohort (yellow star). (d) A direct classifier comparison is made in terms of the mean error rate predicted by each learning curve in (a)-(c).

Error rates predicted by NB and SVM classifiers are similar to those from their LOO error rates of 0.1312 and 0.1333 (Fig 6(b) and 6(c)). In comparison to the learning curves, the slightly lower error rate produced by the validation set is to be expected since the LOO classification is known to produce an overly optimistic estimate of the true error rate [28]. The kNN classifier appears to overestimate error considerably compared to the LOO error of 0.1560, which is not surprising because kNN is a non-parametric classifier that is expected to be more unstable for heterogeneous datasets (Fig 6(a)). Comparison across classifiers suggests that both NB and SVM will outperform kNN as dataset size increases (Fig 6(d)). Although the differences between the mean NB and SVM learning curves are minimal, the 25th and 75th percentile curves suggest that the prediction made by NB is more stable and has lower variance than the SVM prediction.

### Experiment 2: Distinguishing low and high grade cancer in breast histopathology

**Fig 7:**
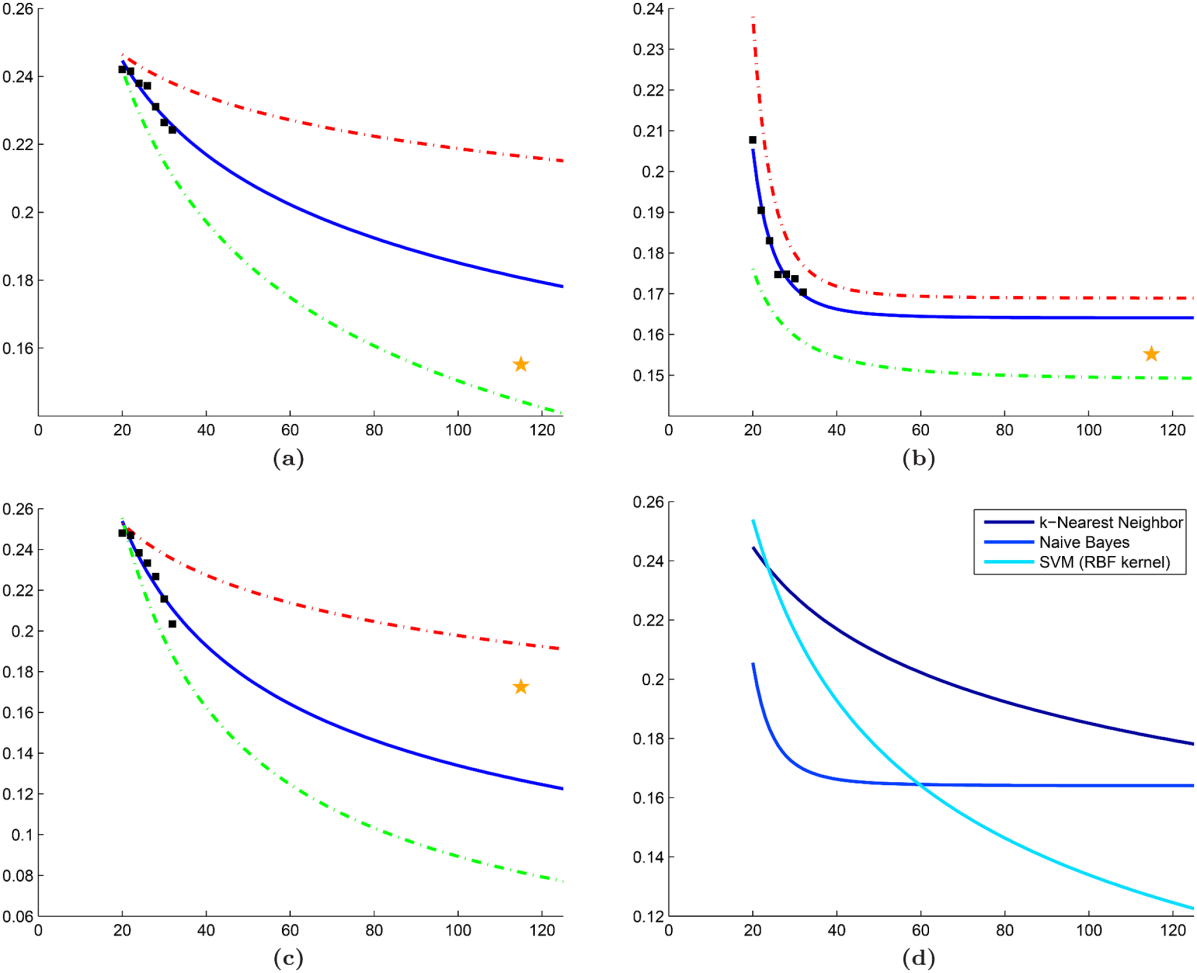
Experimental results for dataset 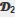. Learning curves (blue line) generated for dataset 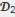 using mean error rates (black squares) calculated from (a) kNN, (b) NB, and (c) SVM classifiers. Each classifier is accompanied by curves for the 25th (green dashed line) and 75th (red dashed line) percentile of the error as well as LOO error on the validation cohort (yellow star). (d) A direct classifier comparison is made in terms of the mean error rate predicted by each learning curve in (a)-(c).

Learning curves from kNN and NB classifiers yield predicted error rates similar to their LOO cross-validation errors (0.1552 for both classifiers) as shown in Fig 7(a) and 7(b). By contrast, while error rates predicted by the SVM classifier are reasonable (Fig 7(c)), they appear to underestimate the LOO error of 0.1724. One reason for this discrepancy may be the class imbalance present in the validation dataset (79 low grade and 37 high grade), since SVM classifiers have been demonstrated to perform poorly on datasets where the positive class (i.e. high grade) is underrepresented [29]. Similar to 𝓓_1_, a comparison between the learning curves reflects the superiority of both NB and SVM classifiers over the kNN classifier as dataset size increases (Fig 7(d)). However, the relationship between the NB and SVM classifiers is more complex. For small training sets, the NB classifier appears to outperform the SVM classifier; yet, the SVM classifier is predicted to yield lower error rates for larger datasets (*n* > 60). This suggests that the classifier yielding the best results for the smaller dataset may not necessarily be the optimal classifier as the dataset increases in size.

### Experiment 3: Distinguishing cancerous and non-Cancerous metavoxels in prostate MRS

**Fig 8:**
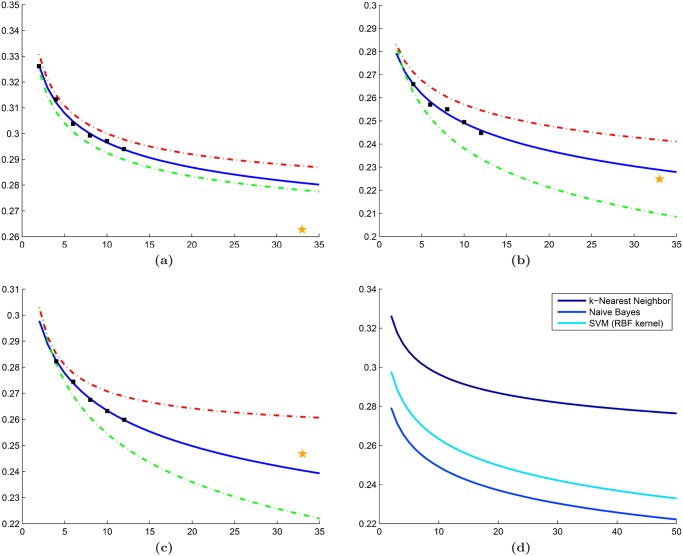
Experimental results for dataset 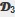. Learning curves (blue line) generated for dataset 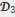 using mean error rates (black squares) calculated from (a) kNN, (b) NB, and (c) SVM classifiers. Each classifier is accompanied by curves for the 25th (green dashed line) and 75th (red dashed line) percentile of the error as well as LOO error on the validation cohort (yellow star). (d) A direct classifier comparison is made in terms of the mean error rate predicted by each learning curve in (a)-(c).

Similar to dataset 𝓓_1_, the LOO error for both the NB and SVM classifiers (0.2248 and 0.2468, respectively) fall within the range of the predicted error rates (Fig 8(b) and 8(c)). Once again, the kNN classifier overestimates the LOO error (0.2628), which is most likely due to the high level of variability in the mean error rates used for extrapolation (Fig 8(a)). While both NB and SVM classifiers outperform the kNN classifier, their learning curves show a clearer separation between the extrapolated error rates for all dataset sizes, suggesting that the optimal classifier selected from the smaller dataset will hold true as even as dataset size increases (Fig 8(d)).

### Comparison with traditional RRS

**Fig 9:**
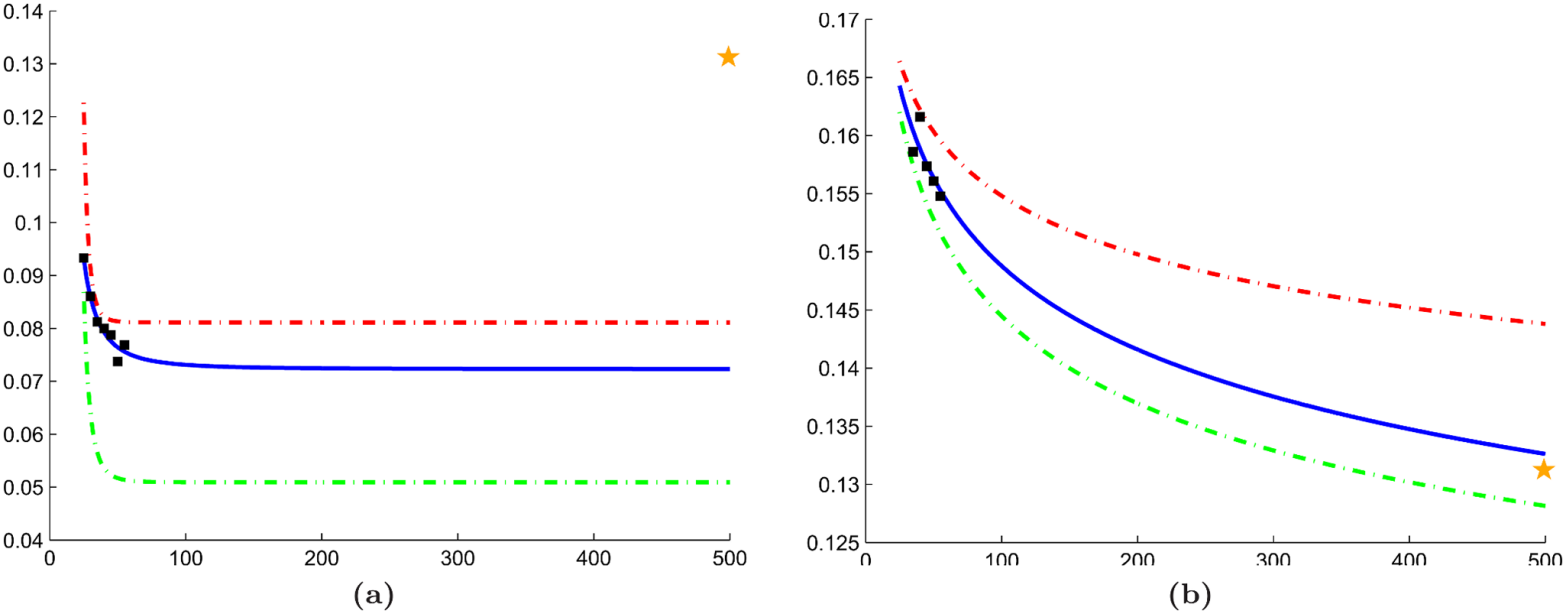
Comparison between traditional random repeated sampling (RRS) and our cross-validated approach. Learning curves generated for dataset 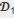 using (a) traditional RRS and (b) cross-validated RRS in conjunction with a Naive Bayes classifier. For both figures, mean error rates from the subsampling procedure (black squares) are used to extrapolate learning curves (solid blue line). Corresponding learning curves for 25th (green dashed line) and 75th (red dashed line) percentile of the error are also shown. The error rate from leave-one-out cross-validation is illustrated by a yellow star.

The quantitative results in Tables 4–6 suggest that employing a cross-validation sampling strategy yields more consistent error rates. In Table 4, traditional RRS yielded a mean IQR ($\bar{\text{IQR}}$ of 0.0297 across all *n* ∈ **N**; whereas our approach demonstrated a lower $\bar{\text{IQR}}$ of 0.0070. Furthermore, a closer look at the learning curves for these error rates (Fig 9) suggests that traditional RRS is sometimes unable to accurately extrapolate learning curves. Similarly, Tables 5 and 6 show lower $\bar{\text{IQR}}$ values for our approach (0.0127 and 0.0140, respectively) than traditional RRS (0.0779 and 0.305, respectively) for datasets 𝓓_2_ and 𝓓_3_. This phenomenon is most likely due to the high level of heterogeneity in medical imaging data and demonstrates the importance of rotating the training and testing pools to avoid biased error rates that do not generalize to larger datasets.

**Table 4:** Mean interquartile range (IQR) demonstrates decreased variability of cross-validated random repeated sampling (RRS) over traditional RRS in dataset 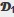.

		n = 25	n = 30	n = 35	n = 40	n = 45	n = 50	n = 55	$\bar{\text{IQR}}$
No CV	P25	0.0833	0.0833	0.0417	0.0417	0.0417	0.0417	0.0833	0.0297
	P75	0.1250	0.0833	0.0833	0.0833	0.0833	0.0833	0.0833	
With CV	P25	–	–	0.1563	0.1579	0.1538	0.1514	0.1522	0.0070
	P75	–	–	0.1609	0.1657	0.1618	0.1596	0.1588	

A comparison between 25th (P25) and 75th (P75) percentile error rates for dataset 𝓓_1_ using traditional RRS (No CV) and our approach (With CV), with mean interquartile range ($\bar{\text{IQR}}$) shown across all *n*. Missing values correspond to error rates that did not achieve significance in the permutation test.

**Table 5:** Mean interquartile range (IQR) demonstrates decreased variability of cross-validated random repeated sampling (RRS) over traditional RRS in dataset 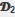.

		n = 20	n = 22	n = 24	n = 26	n = 28	n = 30	n = 32	$\bar{\text{IQR}}$
No CV	P25	0.1818	0.1818	0.1818	0.1818	0.1818	0.1818	0.1818	0.0779
	P75	0.2727	0.2727	0.2727	0.2727	0.2727	0.2727	0.1818	
With CV	P25	0.2456	0.2489	0.2410	0.2347	0.2289	0.2311	0.2190	0.0127
	P75	0.2456	0.2496	0.2494	0.2469	0.2506	0.2498	0.2463	

A comparison between 25th (P25) and 75th (P75) percentile error rates for dataset 𝓓_2_ using traditional RRS (No CV) and our approach (With CV), with mean interquartile range ($\bar{\text{IQR}}$) shown across all *n*. Missing values correspond to error rates that did not achieve significance in the permutation test.

**Table 6:** Mean interquartile range (IQR) demonstrates decreased variability of cross-validated random repeated sampling (RRS) over traditional RRS in dataset 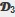.

		n = 2	n = 4	n = 6	n = 8	n = 10	n = 12	$\bar{\text{IQR}}$
No CV	P25	0.2242	0.2113	0.2113	0.2191	0.2294	0.2474	0.0305
	P75	0.2809	0.2577	0.2500	0.2448	0.2448	0.2474	
With CV	P25	0.3176	0.3026	0.2950	0.2873	0.2874	0.2829	0.0140
	P75	0.3345	0.3170	0.3065	0.3003	0.2993	0.2991	

A comparison between 25th (P25) and 75th (P75) percentile error rates for dataset 𝓓_3_ using traditional RRS (No CV) and our approach (With CV), with mean interquartile range ($\bar{\text{IQR}}$) shown across all *n*. Missing values correspond to error rates that did not achieve significance in the permutation test.

### Evaluation of synthetic dataset

**Fig 10:**
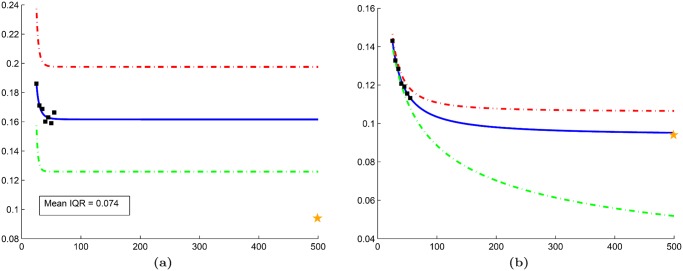
Evaluation of our cross-validated repeated random sampling (RRS) on the synthetic dataset. Learning curves generated for the synthetic dataset using (a) traditional RRS and (b) cross-validated RRS in conjunction with a kNN classifier (k = 3). For both figures, mean error rates from the subsampling procedure (black squares) are used to extrapolate learning curves (solid blue line). Corresponding learning curves for 25th (green dashed line) and 75th (red dashed line) percentile of the error are also shown. The error rate from leave-one-out cross-validation is illustrated by a yellow star.

The reduced variability of cross-validated RRS over traditional RRS is further validated by learning curves generated from the synthetic dataset (Fig 10). Error rates from our approach demonstrate low variability and the ability to create learning curves that can accurately predict the error rate of the validation set (Fig 10(b)). The cross-validated RRS approach yields a mean IQR ($\bar{\text{IQR}}$ = 0.0066) that is an order of magnitude less than traditional RRS ($\bar{\text{IQR}}$ = 0.074).

## Concluding Remarks

The rapid development of biomedical imaging-based classification tasks has resulted in the need for predicting classifier performance for large data cohorts given only smaller pilot studies with limited cohort sizes. This is important because, early in the development of a clinical trial, researchers need to: (1) predict long-term error rates when only small pilot studies may be available and (2) select the classifier that will yield the lowest error rates when large datasets are available in the future. Predicting classifier performance from small datasets is difficult because the resulting classifiers often produce unstable decisions and yield high error rates. In these scenarios, traditional RRS approaches have previously been used to extrapolate classifier performance (e.g. for gene expression data). Due to the heterogeneity present in biomedical imaging data, we employ an extension of RRS in this work that uses cross-validation sampling to ensure that all samples are used for both training and testing the classifiers. In addition, we apply RRS to voxel-level studies where data from both classes is found within each patient study, a concept that has previously been unexplored in this regard. Evaluation was performed on three classification tasks, including cancer detection in prostate histopathology, cancer grading in breast histopathology, and cancer detection in prostate MRS.

We demonstrated the ability to calculate error rates with relatively low variance from three distinct classifiers (kNN, NB, and SVM). A direct comparison of the learning curves showed that the more robust NB and SVM classifiers yielded lower error rates than the kNN classifier for both small and large datasets. A limitation of this work is that all datasets comprise an equal number of samples from each class in order to reduce classifier bias from a machine learning standpoint. However, future work will focus on application to imbalanced datasets where class distribution is based on the overall population (e.g. clinical trials). In addition, we will incorporate additional improvements to the RRS method (e.g. subsampling of testing set as in RIDT) while maintaining a robust cross-validation sampling strategy. Additional directions for future research include analyzing the effect of (a) noisy data on different classifiers [30] and (b) ensemble classification methods (e.g. Bagging) on classifier variability in small training sets.

## Supporting Information

S1 Appendix Description of classifiers. Each experiment in this paper employs three classifiers: k-nearest neighbor (*k*NN), Naive Bayes (NB), and Support Vector Machine (SVM). For ease of the reader we provide a metholodogical summary for each of these classifiers with appropriate descriptions and equations.
(PDF)
