# Supplementary material for: Predicting Classifier Performance with Limited Training Data: Applications to Computer-Aided Diagnosis in Breast and Prostate Cancer
Source: PLoS One. 2015 May 18;10(5):e0117900. doi: 10.1371/journal.pone.0117900 (PMC4436385; doi:10.1371/journal.pone.0117900)
Supplement: S1 Appendix — Each experiment in this paper employs three classifiers: k-nearest neighbor (kNN), Naive Bayes (NB), and Support Vector Machine (SVM). For ease of the reader we provide a metholodogical summary for each of these classifiers with appropriate descriptions and equations. (PDF) [file pone.0117900.s001.pdf]

S1 Appendix: Description of classifiers

Each experiment in this paper employs three classifiers: k-nearest neighbor (kNN), Naive Bayes (NB), and Support Vector Machine (SVM). For all methods described below, let us define a training sample $A \in \mathbf{A}$ and testing sample $B \in \mathbf{B}$ with corresponding feature sets $\mathbf{F}(A)$, $\mathbf{F}(B)$ and ground truth labels $y(A)$, $y(B)$.

k-nearest neighbor classifier

For each testing sample, the kNN classifier identifies the nearest training sample

$$\hat{A}_1 = \underset{A}{\operatorname{argmin}}\, \mathbb{D}(\mathbf{F}(A), \mathbf{F}(B)), \tag{1}$$

where $\mathbb{D}(\cdot, \cdot)$ is a user-specified distance metric. This process is repeated until the k nearest training samples $\{\hat{A}_1, \hat{A}_2, \ldots, \hat{A}_k\}$ have been identified. The class prediction is defined by majority voting across the ground truth labels $\{y(\hat{A}_1), y(\hat{A}_2), \ldots, y(\hat{A}_k)\}$ for all k training samples.

Naive Bayes classifier

The naive Bayes classifier is a simple approach to statistical inference that relies on the application of Bayes' theorem under the assumptions that (1) a sufficient amount of training data is available and (2) its constituent features are independent [1]. For binary classification, let us define the likelihood of observing class ω_1 given feature set $\mathbf{F}$ as

$$P(\omega_1|\mathbf{F}) = \frac{P(\omega_1)p(\mathbf{F}|\omega_1)}{P(\omega_1)p(\mathbf{F}|\omega_1) + P(\omega_2)p(\mathbf{F}|\omega_2)}, \tag{2}$$

where $P(\omega_1)$, $P(\omega_2)$ are the prior probabilities of occurrence of the two classes, and $p(\mathbf{F}|\omega_1)$, $p(\mathbf{F}|\omega_2)$ are the *a priori* class conditional distributions of $\mathbf{F}$. Using all training set samples $\{A : A \in \mathbf{A}, y(A) = \omega_1\}$ in class ω_1, *a priori* distributions $p(F_i(\mathbf{A})|\omega_1)$ are generated for each feature $F_i \in \mathbf{F}$. Due to the relatively small amount of training data used in this paper, kernel density estimation (KDE) is employed to ensure that each $p(F_i(\mathbf{A})|\omega_1)$ is smooth and continuous [2]. Assuming independence between the features allows the distributions to be collapsed such that $p(\mathbf{F}(\mathbf{A})|\omega_1) = \prod_i p(F_i(\mathbf{A})|\omega_1)$. The *a priori* probability $p(\mathbf{F}(\mathbf{A})|\omega_2)$ is similarly generated using samples $\{A : A \in \mathbf{A}, y(A) = \omega_2\}$. Testing sample B is said to be correctly classified if the maximum *a posteriori* decision is equal to the ground truth label, i.e. $y(B) = \operatorname{argmax}_{\omega \in \{\omega_1, \omega_2\}} P(\omega)p(\mathbf{F}(B)|\omega)$.

Support Vector Machine classifier

The SVM classifier [3] operates by projecting training data onto a higher-dimensional space and constructing a hyperplane to maximize the distance between marginal samples in the two object classes. Evaluation is subsequently performed by projecting a testing sample into the same space and ascertaining its location relative to the

hyperplane. In this paper, the projection is defined by calculating the radial basis function (RBF) kernel

$$\Pi(A_1, A_2) = e^{\rho\|\mathbf{F}(A_1)-\mathbf{F}(A_2)\|_2^2} \tag{3}$$

between all pairs of training samples $A_1, A_2 \in \mathbf{A}$, where ρ is a user-defined scaling parameter. The general form of the SVM prediction function is

$$\Theta(B) = \sum_{\gamma=1}^{\tau} \xi_\gamma y(A_\gamma)\Pi(B, A_\gamma) + \mathbf{b}, \tag{4}$$

where $A_\gamma \in \mathbf{A}$ represents a marginal training sample (i.e. support vector), $\mathbf{b}$ is the hyperplane bias estimated over all τ support vectors, and ξ_γ is the slack variable that governs the tradeoff between minimizing training error and maximizing margin [3]. The output of the SVM classifier $\Theta(B)$ represents the distance from testing sample B to the hyperplane, which is determined to be classified correctly if $y(B) = \text{sign}[\Theta(B)]$.

References

1. Duda RO, Hart PE, Stork DG (2001) Pattern Classification. Wiley.

2. John GH, Langley P (1995) Estimating continuous distributions in bayesian classifiers. In: Proceedings of the Eleventh conference on Uncertainty in artificial intelligence. Morgan Kaufmann Publishers Inc., pp. 338–345. URL `http://dl.acm.org/citation.cfm?id=2074196`.

3. Cortes C, Vapnik V (1995) Support-vector networks. Machine learning 20: 273–297.
